# Bioflocculation of pollutants in wastewater using flocculant derived from *Providencia huaxiensis* OR794369.1

**DOI:** 10.1186/s12866-023-03144-w

**Published:** 2024-01-29

**Authors:** Tlou Nelson Selepe, Tsolanku Sidney Maliehe

**Affiliations:** Department of Water and Sanitation, University of Limpopo, Private Bag X1106, Polokwane, 0727 South Africa

**Keywords:** *Providencia huaxiensis* OR794369.1, Optimisation, Bioflocculant production, Flocculating activity, Wastewater treatment, Removal efficiency

## Abstract

**Background:**

Water pollution has become a major environmental and health concern due to increasing population and industrialisation. Microbial flocculants are promising agents for treatment of contaminated water owing to their effectiveness, eco-friendliness, and high biosafety levels. In this study, culture conditions of *Providencia huaxiensis* OR794369.1 were optimised and its bioflocculant was extracted, characterised and used to treat wastewater.

**Results:**

The maximum flocculating activity of 92% and yield of 3.5 g/L were obtained when cultivation conditions were: 3% inoculum size, starch, casein, initial pH of 6, cultivation temperature of 30 ^o^C and 72 h of fermentation. The bioflocculant is an amorphous glycoprotein biomolecule with 37.5% carbohydrates, 27.9% protein, and 34.6% uronic acids. It is composed of hydroxyl, amino, alkanes, carboxylic acid and amines groups as its main functional structures. It was found to be safe to use as it demonstrated non-cytotoxic effects on bovine dermis and African green monkey kidney cells, illustrating median inhibitory concentration (IC_50_) values of 180 and > 500 µg/mL on both cell lines, respectively. It demonstrated the removal efficiencies of 90% on chemical oxygen demand (COD), 97% on biological oxygen demand (BOD) and 72% on Sulphur on coal mine wastewater. It also revealed the reduction efficacies of 98% (COD) and 92% (BOD) and 70% on Sulphur on domestic wastewater.

**Conclusion:**

The bioflocculant was effective in reducing pollutants and thus, illustrated potential to be used in wastewater treatment process as an alternative.

## Background

Water is regarded as a precious substance essential for carbon-based life, development and sustainable ecosystem. Availability of accepted quality of water is one of the major problems faced in the 21st century [1]. Recently, the quality of water is constantly declining due to various anthropogenic activities, increasing population, unplanned urbanisation, and increasing industrialisation. Globally, more than 80% of wastewater is discharged into different waterbodies without proper treatment, making it difficult to achieve the United Nations Sustainable Development Goals such as goal 6 (Clean Water and Sanitation) and goal 14 (Life Below Water) [2]. Furthermore, these two goals seem impossible to achieve by the year 2030 due to the lack of enforcement of pollutant discharge standards and lack of review of the advantages and limitations of the currently used water and/or wastewater treatment technologies.

Flocculation is the pre-treatment technology whereby colloidal particles, which have been destabilised by reducing or eliminating the repulsion forces, agglomerate together into big flocs by flocculating agents called flocculants [3]. The aggregation is mainly dependent on the frequency of collision and affinity between flocculants and colloidal pollutants in aqueous solutions. The two main mechanisms by which pollutants adhere to flocculants are: (1) perikinetic flocculation, which involves agglomeration by induced Brownian motion within colloidal particles and (2) orthokinetic flocculation, which is as the results of the different velocity gradients of colloidal pollutants in aqueous solutions [4].

Flocculants are generally divided into three groups: inorganic (aluminum sulfate and polyaluminum chloride), synthetic organic (polyacrylamide derivatives and polyethylene imine) and naturally occurring (bioflocculants, chitosan and sodium alginate) flocculants [5]. The inorganic and synthetic organic flocculants are predominately utilised in wastewater treatment due to their high efficiencies, ease of operation and cost-effectiveness [6]. However, some of these flocculants are pH sensitive, increase sludge production, promote corrosion to metallic utilities and frequently leave residual metal particles in the treated water. Moreover, they tend to produce harmful monomer residues, which are non-biodegradable and toxic in nature [7, 8]. Hence, the exploration of the natural occurring flocculants such as microbial bioflocculants has gained momentum recently.

Microbial bioflocculants are extracellular polymers secreted by microorganisms such as bacteria, algae and fungi during their growth phase or cell lysis [9]. They are mainly composed of polysaccharides, protein polymers, glycoproteins, lipids, which has functional groups such as hydroxyl and carboxyl groups [10]. These functional groups contribute to the flocculating activities as they provide binding sites to pollutants. Moreover, they determine the type of flocculation mechanisms, which mainly include adsorption, electrostatic patching, polymer bridging, and charge neutralisation [11]. Due to the negative charge of microbial bioflocculants, polymer bridging has been suggested as the main flocculation mechanism. Polymer bridging happens when the biopolymers extend into aqueous solution in a distance greater than that which the colloids` repulsion can exert [12]. Generally, microbial bioflocculants are of great advantage as they are non-toxic, eco-friendly due to their biodegradable nature, pH-insensitive and lack secondary pollution [13]. However, bioflocculant producing strains tend to yield low bioflocculants and this often tend to translate into high production costs. This has been the main bottleneck for bioflocculant production and application at industrial level [14]. Therefore, there is an ongoing search for novel bioflocculant producers with high bioflocculant yields and capabilities from unexplored niches such as marine environment [15].

*Bacillus* species has been the predominant explored bioflocculant producers according to literature [16]. However, other bacterial species such as *Providencia* species have potential to produce desirable bioflocculants. *Providencia* species are Gram-negative rod and have been reported to inhabit heavy metal contaminated environments and to have high absorption capacities for metals such as aluminium, cobalt, copper and palladium [17–19]. Moreover, there have been reports of application of *Providencia* species in bioremediation processes. For instance, *Providencia rettgeri* YL and *Providencia* sp. have been effective in removing heterotrophic nitrogen and chromium, respectively [20, 21]. *Providencia alcalifaciens* 2EA has been utilised in the bioremediation of Pb(II) [22]. Nevertheless, there are no reports of the deployment of *Providencia* species in bioflocculant production. In our previous study, we isolated *Providencia huaxiensis* OR794369.1 from marine fish at Mthunzini Beach sediments in KwaZulu-Natal, South Africa. The bacterium demonstrated potential to effectively produce a bioflocculant with flocculant activity of 66% against kaolin clay suspension (unpublished). In this study, we determined to optimise the culture conditions of *P. huaxiensis* OR794369.1 in order to maximise its bioflocculant yield and to assess the effectiveness of the produced bioflocculant in wastewater treatment. Therefore, the novelty of this study is the use of bioflocculant from *P. huaxiensis* OR794369.1 to bioremediate wastewater.

The aim of this study was to optimise culture conditions of *P. huaxiensis* OR794369.1, characterise and treat wastewater using its bioflocculant.

## Materials and methods

### Chemicals

The chemicals and reagents used in this study were bought from Sigma-Aldrich and Merck (Pty) Ltd, Johannesburg, South Africa. The sea water used in this study was filtered and autoclaved at 121 ^**o**^ C for 15 min at a pressure of 15 psi.

### Culture conditions of ***P. huaxiensis*** OR794369.1

*P. huaxiensis* OR794369.1 is a bioflocculant producer previously isolated from marine fish at Mthunzini Beach sediments in KwaZulu-Natal, South Africa (28°57′S and 31°45′E). In this study, *P. huaxiensis* OR794369.1 was activated on Reasoner´s 2 A agar and cultivated at 37 ^o^C for 24 h. Thereafter, the bacterium was grown on the bioflocculant production medium composing of: glucose (20.0 g), KH_2_PO_4_ (2.0 g), K_2_HPO_4_ (5.0 g), (NH 4) 2SO_4_ (0.2 g), NaCl (0.1 g), CH_4_N_2_O (0.5 g), MgSO_4_ (0.2 g) and yeast extract (0.5 g) in a L of sterilised filtered sea water.

### Optimisation of the culture conditions of ***P. huaxiensis*** OR794369.1

In order to increase the bioflocculant production, growth factors such as inoculum size, nutrients, initial pH, cultivating temperature and incubation time were optimised using one factor at the time method.

### Inoculum size for ***P. huaxiensis*** OR794369.1

The different inoculum sizes were used to evaluate their effect on the bioflocculant production. About 100 mL flasks containing 50 mL of the autoclaved production medium were inoculated with 0.5, 1.0, 1.5 and 2 ml of the isolates to give 1, 2, 3 and 4% (v/v) inoculum sizes, respectively. The inoculums were then incubated for 72 h at 30 ^o^C at the shaking speed of 160 rpm. Afterwards, the broth culture was centrifuged at 13,000 rpm for 15 min and the cell free supernatant was analysed for flocculating activity (FA) [23].

### Analysis of the FA

The free cell supernatant was used to evaluate the FA according to Zwang et al. [24]. Briefly, 2 mL of the cell free supernatant and 3 mL of 1% (w/v) CaCl_2_ were added into 100 mL of kaolin suspension (4.0 g/L, pH 7.0). The mixture was thoroughly shaken at 180 rpm for a minute and then gradually stirred at 50 rpm for 3 min; the solution was then dispensed into a 100 mL measuring cylinder and allowed to precipitate for 5 min. Two millilitres of the clear upper phase layer was aseptically drawn, and its optical density (OD) was measured at 550 nm using a spectrophotometer (Spectro-quant, Pharo 300 Merck, Boston, MA, USA). Two millilitres of sterilised distilled water served as an experimental control. The percentage FA of the samples were measured according to the equation:

%FA = [(C_o_ – C_1_) / C_o_] × 100,

where C_o_ and C_1_ are the OD of the control and the test samples at 550 nm.

### Effect of nutrients, initial pH, cultivation temperature and time course

The effects of carbon and nitrogen substrates were evaluated using the method elucidated by Luo et al. [25]. Briefly, the glucose in the original medium was replaced with 20 g/L of the following carbon sources: fructose, maltose, sucrose, lactose and starch. Thereafter, the flocculating activity was assessed as previously stated. The mixed nitrogen sources (urea, yeast extract and ammonium sulphate) in the original production medium were also replaced with 1.2 g/L of peptone, casein, urea, yeast extract and ammonium sulphate to assess their influence on bioflocculant production [26]. The impact of the initial pH of the medium was determined by firstly adjusting the initial pH of the medium in a range of 4 to 10 using 0.1 M NaOH and 0.1 M HCl. Afterwards, the medium was autoclaved at 121 ^o^C for 15 min; the appropriate inoculum size of *P. huaxiensis* OR794369.1 was inoculated and incubated at 30 °C at the shaking speed of 160 rpm for 72 h. The FA was calculated thereafter [27]. The effect of the culture temperature was measured by growing *P. huaxiensis* OR794369.1 on different temperatures in the range of 20–45 °C. Subsequently, the FA was measured [28]. The relationship between the incubation time, bacterial growth and initial pH of the medium was assessed. The production medium was prepared according to the obtained optimum conditions and inoculated with the optimum inoculum size. Thereafter, the broth culture was drawn and its pH and OD at 550, representing the bacterial biomass, were measured. The culture broth was centrifuged, and the FA was recorded as described earlier. These parameters were analysed every 12 h over a period of 120 h [29].

### Extraction and purification of bioflocculant

The extraction of the bioflocculant from the broth culture was done using solvent extraction. *P. huaxiensis* OR794369.1 was cultured at the obtained optimum culture conditions. The broth culture was centrifuged at 8000 rpm for 30 min. One volume of the sterile distilled water was added into the obtained cell free supernatant and re-centrifuged in order to remove the insoluble materials. Thereafter, two volumes of ethanol was added, and the mixture was allowed to precipitate at 4 °C for 12 h. Afterwards, the precipitate was vacuum-dried, and the crude product was re-dissolved in the sterilised distilled water to give a solution of 1%. In order to purify the bioflocculant, a mixture of chloroform and n-butyl alcohol (5:2 v/v) was pipetted into the bioflocculant solution in a ratio of 2:1 (v/v) and the mixture was left at room temperature for 12 h. The resultant precipitate was collected and vacuum-dried [30].

### Characterisation of the bioflocculant

The extracted bioflocculant was subjected to various characterisation methods to identify its properties. The composition of the bioflocculant in terms of the percentage polysaccharides, proteins and uronic acids within the bioflocculant was evaluated by phenol-sulfuric acid, Bradford and carbazole-sulfuric acid assays [31, 32]. Scanning electron microscopy (SEM) was utilised to ascertain the morphology of the bioflocculant [33]. The Fourier transform infrared spectroscopy (FTIR) analysis was employed to evaluate the active groups responsible for bioflocculation. Further characterisation were performed using an energy dispersive X-ray spectrometer, which was used to quantitatively identify elemental composition of the bioflocculant [34]. The biosafety of the bioflocculant was evaluated by determining its cytotoxic effect on the African green monkey kidney (Vero) and Bovine dermis using 3-(4,5-dimethylthiazol-2-yl)-2,5-diphenyl tetrazolium bromide (MTT) assay [35].

### Effect concentration, cations and initial pH on the flocculating activity

The impact of concentration on FA was determined by varying the bioflocculant concentrations in the range of 0.2–0.8 mg/mL. Thereafter, the FA was measured as previously stated. The influence of cations on flocculation was determined by replacing the 1% CaCl_2_ solution with NaCl, LiCl, KCl, MnCl_2_, MgCl_2_ and FeCl_3_. Afterwards, the flocculation assay was carried-out and the FA was recorded. The effect of initial pH of kaolin solution (4 g/mL) on FA was investigated by adjusting the initial pH between 4 and 10 using 0.1 M HCl and 0.1 M NaCl. Thereafter, the bioflocculant and appropriate cation were added into the mixture and the FA was determined [36].

### Wastewater treatment using the bioflocculants

The bioflocculant was applied to treat wastewater from a coal mine wastewater treatment plant in KwaZulu-Natal and Erwat Wastewater Treatment Plant in Gauteng, South Africa. The pH of the wastewater were adjusted to pH 4 using 0.1 M HCl and 0.1 M NaCl. Thereafter, the wastewater was treated with the bioflocculant, chemical flocculants, such as aluminium sulphate and ferric chloride. The removal efficiencies of the flocculants on the chemical oxygen demand (COD), biological oxygen demand (BOD) and sulphur were measured using test-kits, following the manufacturer`s guidelines. The removal efficiency (RE) of each flocculant was expressed in percentage as:

%RE = [Xo - X / Xo] x 100,

whereby Xo and X represent the values obtained before and after treatment [37].

### Data analysis

All data experiments were performed in triplicate and expressed as mean values. The standard deviations were calculated. A one-way analysis of variance (ANOVA) was used using the Graph Pad Prism version 8. A significance difference level of *p* ˂ 0.05 was vied as statistically significant.

## Results and discussion

### Optimisation of medium composition and culture conditions

#### Inoculum size of ***P. huaxiensis*** OR794369.1

Table 1 illustrated the impact of bacterial inoculum size on the production of the bioflocculant. At an inoculum size of 3% (v/v), maximum flocculating activity of 85% was attained. An increase in the inoculum size from 3% led to an insignificant drop in bioflocculant production. The inoculum sizes less than 3% prolonged the lag phase *P. huaxiensis* OR794369.1, consequently leading to low bioflocculant production whereas the inoculum size greater than 3% led to niche overlap of *P. huaxiensis* OR794369.1, which might have resulted in the observed insignificant drop in the bioflocculant production [38]. Therefore, at the inoculum size of 3%, *P. huaxiensis* OR794369.1 had maximum ability to grow and produce bioflocculant efficiently. Thus, the inoculum size of 3% was used in the following experiments. Literature does illustrate that bioflocculant-producer with inoculum size ranging from 1 to 5% are of noteworthy.

**Table 1:** Effect of inoculum size, nutrients, initial pH and temperature on bioflocculant production

Inoculum size (%)	%FA	Carbon source	%FA	Nitrogen source	%FA	pH	%FA	Temperature (^o^C)	%FA
1	60 ± 3.8^a^	Starch	93 ± 5.3^a^	Peptone	71 ± 8.9^a,b^	4	68 ± 3.2^a^	20	73 ± 2.1^a,b^
2	70 ± 3.1^a^	Glucose	66 ± 6.2^b^	Urea	74 ± 3.8^b^	5	70 ± 4.2^a^	25	78 ± 2.3^b^
3	85 ± 6.2^b^	Maltose	90 ± 4.3^a^	Yeast extract	78 ± 9.0^b^	6	92 ± 0.3^c^	30	92 ± 0.2^c^
4	83 ± 9.0^b^	Sucrose	63 ± 5.8^b^	Ammonium sulphate	79 ± 7.1^b^	7	80 ± 3.2^b^	35	80 ± 0.8^b^
		Fructose	85 ± 7.5^a^	Casein	83 ± 4.7^b^	8	81 ± 1.0^b^	40	76 ± 2.1^b^
		Lactose	37 ± 1.2^c^	Mixed nitrogen sources (yeast extract, urea and ammonium sulphate	66 ± 6.2^a^	9	73 ± 1.3^a,b^	45	62 ± 1.2^a^
						10	45 ± 2.3^a^		

The superscripts (a, b and c) show the statistically significant (*p* < 0.05); similar letters illustrate no statistical difference (*p* > 0.05)

#### Effect of nutrients on bioflocculant production by ***P. huaxiensis*** OR794369.1

Table 1 displays the impact of different medium composition on bioflocculant production by *P. huaxiensis* OR794369.1. The low bioflocculant production was observed when lactose was utilised as the carbon source, suggesting that *P. huaxiensis* OR794369.1 lacked lactase to breakdown lactose for bioflocculant production. However, *P. huaxiensis* OR794369.1 was capable of effectively metabolising a wide range carbon sources such as starch, maltose and fructose for bioflocculant production yielding equal to or above 85% of flocculation. Due to the cost effectiveness and availability, starch was used as a carbon sources in the subsequent experiments. A similar phenomenon where starch was effectively utilised as the most suitable carbon source for the production of a bioflocculant produced was recorded on *Aspergillus parasiticus* [39]. In addition, *P. huaxiensis* OR794369.1 was found to have the ability to effectively metabolise all the utilised nitrogen sources for bioflocculant production, giving flocculating activities above 70% (Table 1). Casein was utilised as a nitrogen source in subsequent experiments.

#### Effect of initial pH of the culture medium

The effect of the initial pH on the culture medium on the bioflocculant production by *P. huaxiensis* OR794369.1 was evaluated, and the results are displayed in Table 1. There was a significant increase (*p* < 0.05) observed in the bioflocculant production with the increase in pH from pH 4 up to pH 6, which yielded maximum bioflocculant, with the activity of 92%. This suggested that the initial pH of 6 was conducive for *P. huaxiensis* OR794369.1 to effectively activate enzymatic reactions and maintenance of optimum oxidation-reduction potential, consequently enabling nutrient assimilation and bioflocculant production [40]. However, an increase in the pH from pH 6 to neutral and alkali resulted in the decline of bioflocculant production, implying the negative effect on the electric status of the bacterium. The lowest bioflocculant biosynthesis was observed when the initial pH of 10 was utilised, yielding 45% of flocculating activity. The results were comparable with those obtained by Aljuboori et al. [41], whereby the bioflocculant produced by *Aspergillus niger* sp had shown high bioflocculant production at pH 6.

#### Effect of culture temperature

The influence of culture temperature on bioflocculant production *P. huaxiensis* OR794369.1 is illustrated in Table 1. The temperature increased from 20 to 30 ^o^C led to a significant increase (*p* < 0.05) in the bioflocculant production by *P. huaxiensis* OR794369.1. The highest bioflocculant production as revealed by the flocculating activity was observed at 30 ^o^C. As the culture temperature was raised above 30 °C, the decline in the bioflocculant production was recorded. The lowest bioflocculant production was observed at the highest culture temperature used (45 ^o^C), giving the flocculation rate of 62%. This drop might be due to denaturation of enzymes involved in the bioflocculation production. On the other hand, the low production in the low temperatures (˂ 30 ^o^C) might have been induced hibernation, consequently leading to poor enzymatic activities [42]. The results were contrary to those reported by Giri et al. [43], whereby the bioflocculant producing strain achieved optimal bioflocculant production at the high temperature (40 °C).

#### Time effect on bioflocculant production, medium pH and bacterium growth

**Fig. 1:**
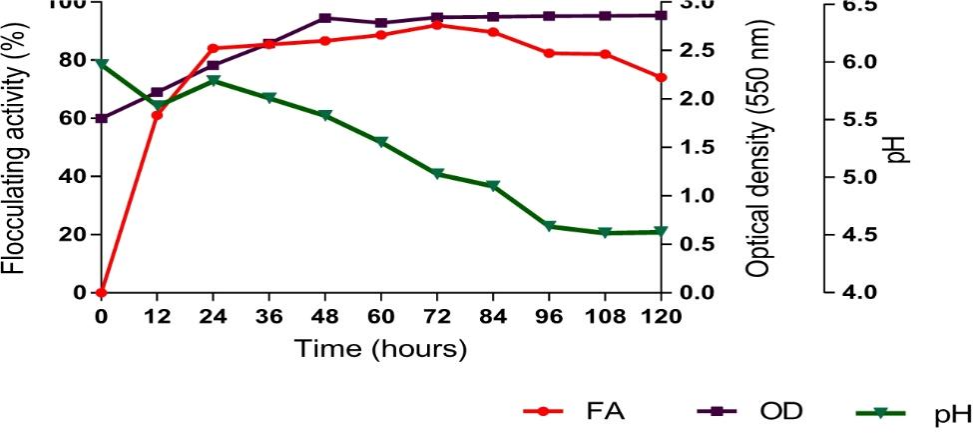
Relations between *P. huaxiensis* OR794369 growth rate, pH and bioflocculant production

Figure 1 illustrates the growth curve, bioflocculant production and effect of growth on the initial pH of the medium. There was an increase in the OD_600_ with the increase in time up until 48 h, implying that the bacterium was in its exponential growth phase. After 48 h onwards, the OD_600_ became constant, implying that the bacterium entered stationary and death phases due to the depletion of nutrients in the medium. It was also noted that the bioflocculant production was along with cell growth up 72 h, signaling that the bioflocculant production influenced by the cell growth. The bioflocculant production peaked at 72 h and revealed the maximum flocculation activity of 92%. After 72 h of cultivation, the bioflocculant production decreased monotonically, and the decline was owed to cell autolysis and the decrease in enzymatic activity [44]. The initial pH of the medium (pH 6) drastically decreased to pH 4.5 at the end of the cultivation time. The decrease in pH was thought to be attributable to the production of acidic components as the results of the metabolism of starch and cacein in the medium.

#### Bioflocculant yield of ***P. huaxiensis*** OR794369.1

The obtained purified bioflocculant was 3.5 g from 1 L of the culture broth. The yield is higher yield as compared to bioflocculant previously extracted from *Virgibacillus* sp *Rob*, *Bacillus firmus*, *Enterobacter clocoae* and *Proteus mirabilis* which revealed yields less than 3 g/L [28, 45]. The moderate yield obtained in this study suggested the potential and economic importance of *P. huaxiensis* OR794369.1 in bioflocculant production at the industrial level.

#### Characterisation of the bioflocculant

**Fig. 2:**
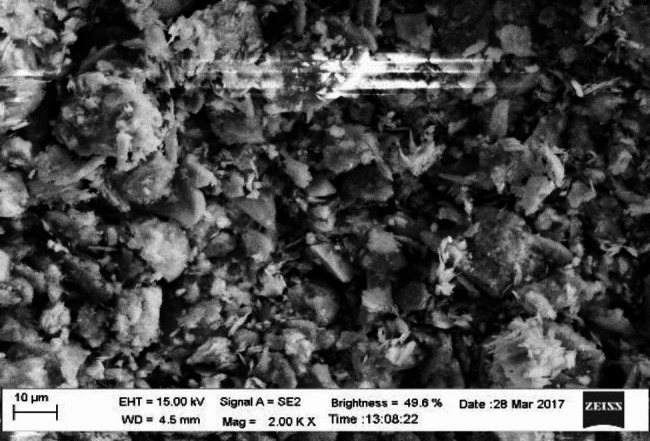
SEM image of the bioflocculant structure from *P. huaxiensis* OR794369.1

The SEM spectrum revealed the bioflocculant to have an amorphous structure (Fig. 2). Moreover, the bioflocculant was whitish in colour. The bioflocculant structural configuration may contribute to its profound flocculating efficiency [46].

#### Chemical composition of bioflocculant

The chemical composition of the bioflocculant from *P. huaxiensis* OR794369.1 revealed the presence of carbohydrates (37.5%), (27.9%) protein, and (34.6%) uronic acids, respectively. The revealed bioflocculant`s composition confirmed it to be a glycoprotein molecule. In addition, the high uronic acid content signal the potential presence of carboxyl groups, which are binding sites for pollutants [47].

#### Elemental composition of the bioflocculant

The EDX analysis showed the mass ratio of elements as indicated in Table 2. The EDX analysis revealed carbon (49.4%) and oxygen (43.7%) as the predominant elements whereas potassium and Sulphur were relatively the lowest with 0.2%. The presence of carbon, oxygen and nitrogen affirmed the bioflocculant to be a glycoprotein molecule. Moreover, the presence of these elemental constituents were assumed to account for the stability and flexibility of the bioflocculant [37].

**Table 2:** Elemental composition of the bioflocculant from *P. huaxiensis* OR794369.1

Element	Occurrence (% (w/w)
Carbon	49.4
Oxygen	43.7
Phosphorus	1.9
Calcium	1.7
Chlorine	0.9
Nitrogen	0.8
Magnesium	0.6
Potassium	0.2
Sulphur	0.2

#### Functional groups of the bioflocculant from ***P. huaxiensis*** OR794369.1

**Fig. 3:**
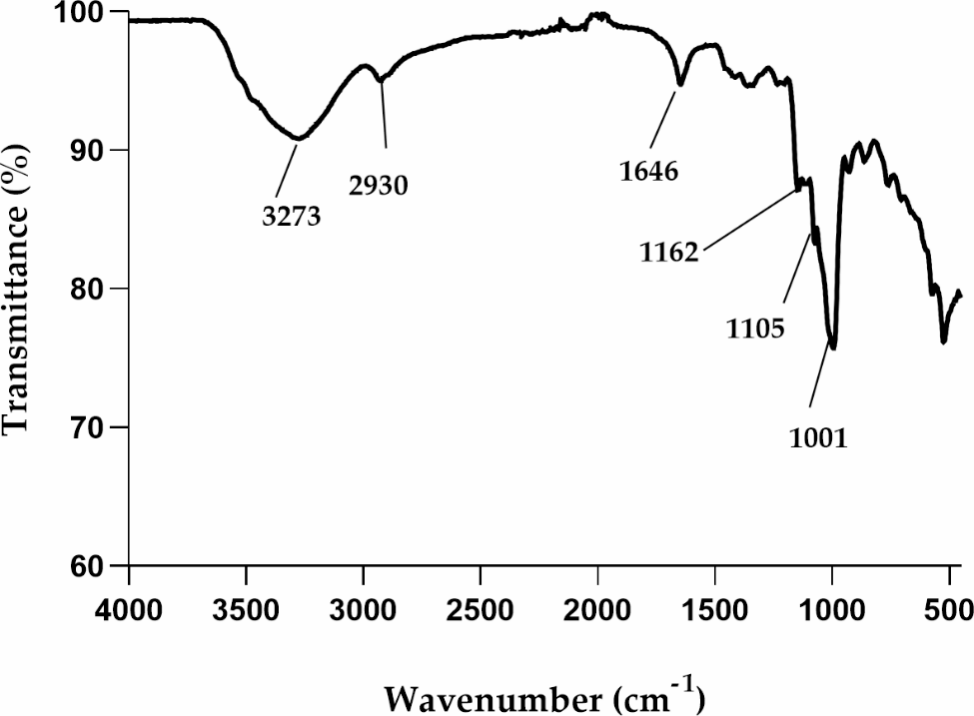
FTIR spectra of the bioflocculant from *P. huaxiensis* OR794369.1

FTIR spectrum revealed the functional groups of bioflocculant from *P. huaxiensis* OR794369.1. It displayed a broad peak at around 3273 cm^− 1^ characteristics for hydroxyl and amino groups. The peak at 2930 cm^− 1^ was indicative of C-H stretch, representing alkanes. Absorption of primary amines was indicated by the peak at 1646 cm^− 1^. Furthermore, absorptive peaks at 1162 cm^− 1^ and 1001 cm^− 1^ displayed C-O stretch, representing tertiary alcohols and carboxylic acid, respectively (Fig. 3). The presence of numerous functional groups in the bioflocculant provide more binding sites for the pollutants, resulting in enhancement of bioflocculation process [44]. Additionally, the presence of hydroxyl, amino and amines groups confirm the bioflocculant as a glycoprotein molecule. These results were in consistency with the observations of Xia et al. [28], whereby the bioflocculant produced by *Proteus mirabilis* TJ 1 revealed to have carboxyl, hydroxyl and amino groups as preferred groups for the flocculation process.

#### Biosafety of the bioflocculant from ***P. huaxiensis*** OR794369.1

The bioflocculant was found to be non-cytotoxic against bovine dermis and African green monkey kidney (Vero) cells, illustrating the IC_50_ of 180 and > 500 µg/mL on both cell lines, respectively. The bioflocculants are considered significantly toxic when there is IC_50_ < 30 µg/mL [48]. Therefore, the attained results confirmed the bioflocculant to be non-cytotoxic, implying that the bioflocculant can be safely utilised in various industrial fields within the appropriate concentrations. The results were in accordance with those obtained by Selepe et al. [49], in which the bioflocculant from *Chrobactrum oryzae* AB84113 showed no cytotoxic effect.

#### Effect of concentration on bioflocculation

The flocculation efficiency increase insignificantly (*p* > 0.05) with an increase in bioflocculant concentration up until 0.4 mg/mL. Maximum bioflocculation efficiency of 87% was obtained at 0. 4 mg/mL. Moreover, there was an insignificant decrease (*p* > 0.05) in bioflocculation rate when concentrations above 0.4 mg/mL were used (Table 3). This can be attributed to the oversaturation of bioflocculant molecules on the kaolin binding sites, which might have led to re-introduction of repulsion forces between the bioflocculant and kaolin particles. However, although 0.4 mg/mL gave insignificantly higher activity (*p* > 0.05), 0.2 mg/mL, which yielded 83%, was utilised in this study for economic reasons. Furthermore, 0.2 mg/mL enabled bridge formation between the bioflocculant and kaolin particles effectively the same way as those of higher concentrations [50].

**Table 3:** Effect of concentration, cations and pH on FA of the bioflocculant

Concentration (mg/mL)	%FA	Cations	%FA	pH	%FA
0.2	85 ± 3.7^a^	No cation	49 ± 2.2^b^	4	90 ± 3.3^c^
0.4	87 ± 2.8^a^	Na^+^	88 ± 4.6^a^	5	88 ± 2.1^a,c^
0.6	85 ± 4.6^a^	Li^+^	87 ± 2.4^a^	6	84 ± 0.8^a^
0.8	85 ± 3.5^a^	K^+^	86 ± 4.6^a^	7	85 ± 3.7^a^
		Ca^2+^	87 ± 2.8^a^	8	78 ± 2.4^a,b^
		Mg^2+^	86 ± 2.1^a^	9	77 ± 3.3^b^
		Mn^2+^	84 ± 5.9^a^	10	71 ± 0.7^b^
		Fe^3+^	83 ± 5.3^a^		

The superscripts (a, b and c) show the statistically significant (*p* < 0.05); similar letters illustrate no statistical difference (*p* > 0.05)

#### Effect of cations and initial pH on FA

Table 3 depicts the effect of various cations on the flocculating efficiency of the purified bioflocculant. All tested cations significantly promoted flocculating efficiency of the bioflocculant, revealing above 80% activity. This implied that the bioflocculant is cation dependent. The cations were able to facilitate the flocculation rate by neutralising the charge of the bioflocculant and the kaolin particles, thereby enabling formation of bridges between the bioflocculant and kaolin particles [38]. The findings in this study aligned with those obtained in other studies, whereby bioflocculants were cation-dependent [51]. The bioflocculant further revealed to be highly effective in acidic, neutral and weak alkali conditions. The peak flocculating activity of 90% was recorded at pH of 4 and the lowest activity (71%) was reported at a high pH of 10 (Table 3). It was noted that the increase in OH^−^ concentration as the pH was increased, possibly increased the electrostatic repulsion between the bioflocculant molecule and the kaolin particles, consequently resulting in the significant decline (*p* ˂ 0.05) in the flocculating activity. Nevertheless, the effectiveness of the bioflocculant over a wide pH range is of economic advantage as there is no need to adjust the wastewater. The findings also agree with those obtained by Zhang et al. [24], in which a bioflocculant from *Ruditapes philippinarum*, demonstrated high flocculating efficiency over a wide pH range.

#### Wastewater treatment by the bioflocculant

The removal efficiencies of the bioflocculant from *P. huaxiensis* OR794369.1 was comparatively the same (*p* > 0.05) to those of aluminum chloride and ferric chloride on the COD and BOD of both mine and domestic wastewater. The bioflocculant demonstrated 90 and 97% reduction efficiencies on the COD and BOD of the mine and 98 and 92% on COD and BOD of domestic wastewater, respectively. However, it demonstrated significantly better reduction efficiencies on sulphur in both wastewater in comparison to aluminum chloride and ferric chloride. It gave 72% reduction efficacy on mine wastewater and 72% on the domestic wastewater (Table 4). The profound reduction ability of the bioflocculant on the tested pollutants was accredited to the active functional components of the bioflocculant, which are able to bind and remove the pollutants in wastewater. Therefore, the findings in this study support the use of microbial flocculants for treatment of wastewater as a viable alternative in relation to the predominately utilised chemical flocculants. Our findings were consistent with the results obtained by Selepe et al. [52] and Ugbenyen et al. [53], where the bioflocculants effectively reduced contaminants in wastewater.

**Table 4:** Percentage removal efficiencies of flocculants

Flocculants	Coal Mine Wastewater			Domestic Wastewater		
	COD	BOD	Sulphur	COD	BOD	Sulphur
Bioflocculant	90 ± 2.3^a^	97.7 ± 6.2^a^	72 ± 0.4^a^	98 ± 1.5^a^	92 ± 5.3^a^	70 ± 2.3^a^
Aluminium sulphate	98 ± 0.5^a^	96 ± 0.9^a^	94 ± 2.6^b^	97 ± 1.3^a^	82 ± 3.2^a^	90 ± 3.4^b^
Ferric chloride	94 ± 3.5^a^	97 ± 4.3^a^	97 ± 7.4^b^	99 ± 2.3^a^	86 ± 2.1^a^	89 ± 4.1^b^

The superscripts (a,b) show the statistically significant (*p* < 0.05); similar letters illustrate no statistical difference (*p* > 0.05)

## Conclusion

The optimisation of the medium and culture conditions of *P. huaxiensis* OR794369.1 led to 26% improvement in bioflocculant production. The bioflocculant revealed to be a glycoprotein composing of diverse functional groups. Furthermore, it revealed to be safe to use as it illustrated non-cytotoxic effects. In addition, the biofllocculant exhibited excellent removal efficiencies on the tested parameters in coal mine and domestic wastewater. The high flocculating activity was attributed to the observed functional groups. The bioflocculant illustrated potential applicability in wastewater treatment process as an alternative to chemical flocculants. For further studies, the genes and metabolic pathways involved during bioflocculant production by *P. huaxiensis* OR794369.1. Furthermore, the exploration of the mode of flocculation by the bioflocculant ought to be evaluated.

## Data Availability

The bacterium analysed in this study is available from Genbank (https://submit.ncbi.nlm.nih.gov/subs/?search=SUB13968371). The accession number of the bacterium is provided in this manuscript. The other datasets used during this study are available from the corresponding author on request.

## Abbreviations

*P. huaxiensis*: *Providencia huaxiensis*
FA: flocculating activity
OD: optical density
SEM: scanning electron microscopy
FTIR: Fourier transform infrared spectroscopy
COD: chemical oxygen demand
BOD: biological oxygen demand
